# Change of risk behaviour in young people – the effectiveness of the trauma prevention programme P.A.R.T.Y. considering the effect of fear appeals and cognitive processes

**DOI:** 10.1186/s12889-022-12918-2

**Published:** 2022-03-26

**Authors:** Michael Koehler, Thomas Brockamp, Sebastian Bamberg, Tina Gehlert

**Affiliations:** 1 Institute of Health and Nursing Science, Charité – Universitätsmedizin Berlin, Augustenburger Platz 1, 13353 Berlin, Germany; 2 Department of Trauma and Orthopaedic Surgery, University of Witten/ Herdecke, Cologne-Merheim-Medical-Center (CMMC), Ostmerheimer Street 200, 51109 Cologne, Germany; 3 Bielefeld University of Applied Sciences and Arts, Interaktion 1, 33619 Bielefeld, Germany; 4 German Insurer’s Accident Research, Wilhelmstraße 43 / 43G, 10117 Berlin, Germany

**Keywords:** P.A.R.T.Y. program, Risk behaviour, Prevention, Youth, Fear appeals, Cognitive beliefs

## Abstract

**Background:**

The purpose of the present study was to examine the effectiveness of the injury awareness and prevention programme P.A.R.T.Y. (Prevent Alcohol and Risk-Related Trauma in Youth) in Germany. On a designated P.A R.T.Y. day, school classes spend a day in a trauma hospital experiencing the various wards through which a seriously injured person goes. A further goal of the study was to reveal indications of the programme’s mechanism of action by testing theory-based impact models of fear appeals and cognitive beliefs.

**Methods:**

In a quasi-experimental longitudinal study with three measurement times the participants of 19 P.A.R.T.Y. days (*n* = 330), as well as pupils who did not attend the programme (*n* = 244), were interviewed with a standardised questionnaire. They reported risk behaviour, feelings of threat and cognitive beliefs about road traffic. The data were analysed using a meta-analytical approach to estimate an average effect size across the different P.A.R.T.Y. days. Path models were used to identify possible mechanisms of action.

**Results:**

For most of the parameters, small positive effects could be proven immediately after the P.A.R.T.Y. intervention. However, after four to 5 months only one statistically significant effect was found. Using path analytical models, important predictors for behavioural changes (e.g. self-efficacy) could be identified. But for these predictors no or only short-term effects were observed in the meta-analysis.

**Conclusions:**

Fear appeals as used primarily in the P.A.R.T.Y. programme appear to cause behavioural changes only to a limited extent and only in the short-term, especially if the strengthening of psychosocial resources is not given sufficient consideration. The participants must also cognitively process the experiences in the hospital. Accordingly, consideration should be given to how the P.A.R.T.Y. program could be adapted to complement the fear appeal with cognitive components.

**Supplementary Information:**

The online version contains supplementary material available at 10.1186/s12889-022-12918-2.

## Background

Injuries are the most common cause of death among teenagers and young adults worldwide. Road traffic accidents are a significant factor for the high incidence of injuries [1, 2]. Preventing risk-taking behaviour could substantially reduce the risk of unintentional injuries and their fatal consequences [3]. Frequent risk-taking behaviour among young drivers include, but are not limited to driving under influence of alcohol [4, 5], speeding [6, 7] or distraction, especially by using a mobile phone [8, 9]. In order to address this problem, there are numerous behavioural prevention interventions. This tend to deploy psycho-educative approaches. Often no statements can be made about the effectiveness of these measures, as there are no studies available. Existing evaluation studies indicate that many of these educational programs show little or no effects on behaviour change, especially in adolescents and young adults [10–13]. Many of them use dissuasive or frightening content, which is supposed to change the behaviour of adolescents and young adults in the future [13, 14].

The prevention programme P.A.R.T.Y. (Prevent Alcohol and Risk Related Trauma in Youth) is another example of such an intervention. It is a one-day injury awareness and prevention program for youth. A school class spends a day in a trauma centre and experiences the various wards of a seriously injured patient. The P.A.R.T.Y. programme was developed in Canada and has been carried out there for more than 30 years. The programme development and implementation was motivated by the fact that injuries are the leading cause of death in young people and that most of these injuries are predictable as well as preventable [15].

Hence, it was also introduced in many other countries, such as Australia, New Zealand and the United States. The German Society for Traumatology and the Academy of Traumatology GmbH have adapted the concept to German standards. Since 2012, it has been implemented in over 40 trauma centres in Germany. The objective of the programme is to change the behaviour of young people by illustrating the negative consequences of traffic accidents. It is intended to increase awareness for serious consequences of risk behaviour and aims to reduce accidental injuries and deaths in this age group. It is based on learning by means of real people’s experiences through vividness and emotions [15].

Initially, the participants receive two half-hour lectures, each on the topics of trauma and prevention, usually held by a trauma surgeon (trauma) and a police officer (prevention). Afterwards, the pupils pass through the individual wards that a severely injured patient is likely to go through. These are the ambulance, the emergency ambulance/trauma room, the intensive care unit and the normal ward. A so-called P.A.R.T.Y. guide – a hospital staff from the medical or nursing service – accompanies the pupils. The participants get an idea of how a seriously injured patient is cared for in each ward. If possible, they can talk to the patients there. Afterwards, the participants visit physiotherapy and see how tedious and difficult rehabilitation can be after a serious injury. Finally, there is a 20-min talk with a former seriously injured patient. The P.A.R.T.Y. day ends with a joint reflection on the day.

Previous evaluation studies find short-term programme effects (1–2 weeks after implementation) on knowledge and attitudes [16–21] as well as medium-term effects (6–12 months after implementation) on self-reported risk behaviour [19, 21, 22]. Furthermore, there seem to be long-term effects (up to 44 months after implementation) on traffic offences, injuries and deaths [23, 24]. The study designs used in these previous evaluation studies range from simple pre-post-test designs to quasi-experimental pre-post-test designs controlling the influence on possible group differences e.g. by using propensity scores. However, the most studies use rather simple study designs. In the majority of studies, simple before/after comparisons without a control group were used (e.g. [18, 19]). Furthermore, positive changes reported are mostly based on measuring the percentage of more “correct” or “better” responses after P.A.R.T.Y. compared to the time before the intervention. Therefore, it is unclear to what extent the participants’ behaviour was actually reached. It must be considered that the described limitations of the deployed study designs used reduce the internal validity of the findings. The controlled studies are also limited, as they are, for example, by retrospective [22], not randomized, but matched control groups [23], or the target group consisting exclusively of adolescents who have committed criminal offenses [24]. There is no study using a randomized control trial design. Furthermore, most studies are outcome evaluations measuring the effects after the programme. There is no study investigating the supposed effect mechanisms of the programme, namely fear appeal. These design components should be considered in the present study to get more precise information about the effectiveness and effect mechanisms of the P.A.R.T.Y. programme.

The contents of P.A.R.T.Y. indicate that the programme attempts to change the behaviour of young people, particularly through fear appeals. Whereas most practitioners seem to judge fear appeals as very effective for changing behaviour, researchers are more sceptical. This scepticism seems appropriate, because after all, some meta-analyses support this attitude [25, 26]. However, even within the scientific community, one does not always seem to be so critical of the behavioural impact of fear appeals. Thus, Kok et al. discuss and criticise the often-false conviction regarding the effect of fear appeals on behaviour and the way in which they are dealt with in politics but also in science [27]. They argue that self-efficacy in particular needs to be taken into account. That holds true for the implementation but also for the scientific evaluation of the effectiveness of health promotion interventions based on fear appeals. Perceived threat through fear appeals only leads to the desired behaviour together with high self-efficacy (see also [28]). They argue based on findings from studies on the effectiveness of scary pictures on cigarette packets and do not consider the positive effects in these studies convincing, since these studies did not take into account the extent of self-efficacy [27]. Numerous comments were written in response within a very short time period [29–35]. These papers criticize Kok and colleagues for taking a one-sided view of the existing literature and for providing evidence that fear appeals may well have a bearing on health behaviour change. To pour oil on troubled water, the initiators (originally [36]) of this debate tried to find a consensus in a further contribution, how to work together on how to deal with fear appeal in science to achieve the goal of designing effective behaviour change interventions [37]. In the end, they promote working together to identify the strongest determinants of a desirable behaviour and the most effective methods to change them as well as ways to apply those methods in the light of health communication constraints.

The debate presented thus shows that the effectiveness of fear appeals may depend on certain framework conditions and circumstances [29]. Finally and importantly, the complexity of the interaction of different factors that determine behaviour must be taken into account. Risk perception or disgust alone may not be sufficient to bring about successful change [37].

Therefore, the first objective of this study was to determine the effectiveness of the fear appeals-based P.A.R.T.Y. programme in Germany for changing risk behaviour in traffic. The second objective was to investigate the impact mechanism by which the behaviour change is supposed to be achieved. Neither the original programme description nor the previous evaluation studies provide a theory-based impact model that describes the process of behaviour change. Especially the effect of fear appeal is of interest, as participants of the programme are supposed to be convinced by negative consequences of traffic accidents.

### Determining P.A.R.T.Y. programme’s potential impact mechanisms

So far, there is no programme theory that explains the intended process of behaviour change underlying the P.A.R.T.Y. programme. However, a well-formulated theory is necessary to understand the effectiveness of a prevention programme and to further improve it [28]. From the programme itself it is evident, that fear appeal plays a role. The scientific debate around fear appeal reveals that effectiveness of fear appeals might depend on certain framework conditions and circumstances. Therefore, cognitive factors were considered that might act within the programme and interact with fear appeal. The following theoretical models were considered.

### Fear appeals and threat feelings

Fear appeals aim at motivating people to omit disapproved behaviour or to show other approved behaviour by conveying negative or painful consequences of this disapproved behaviour [38]. In road safety campaigns, fear appeals, which often combine haunting stories and shocking pictures or films, are used because they encourage people to focus their attention on otherwise boring or unattractive topics (e.g. wearing a bicycle helmet).

Despite intensive research on the effectiveness of fear appeals over the last few decades, the presented debate shows that messages intended to generate emotions such as feelings of fear and threat are not easy to handle. Findings suggest that fear appeals can activate opposing mechanisms, resulting in an inconsistent picture of the effectiveness of fear appeals [38–41]. Witte describes these mechanisms in the Extended Parallel Processing Model [38]. It is based on Leventhal’s Parallel Response Model [42] and Roger’s Protection Motivation Theory [39]. It is a frequently used model for studying and explaining the effect of fear appeals on behaviour. The model describes how the interaction of perceived threat and perceived efficacy of controlling the threat can result in different behavioural decisions. In a first evaluation process the extent to which a person perceives the fear appeal as a threat determines whether a feeling of fear occurs at all or no reaction is to be expected. Only if the message is perceived as a serious threat, it triggers fear, which in turn leads to an impulse to act. In this second process, a person’s confidence in being able to actively counter the threat actively, determines the nature of the action itself. Thus, the fear of a perceived threat can trigger adaptive behaviour only when the perceived expectation of effectiveness is high. In this case, the model predicts self-protective actions to control the threat, e.g. reducing risk-taking behaviour. However, if the level of effectiveness is perceived to be low, maladaptive behaviour occurs. Those defensive reactions are not aimed at reacting to the threat, but to control the fear itself. Then the negative feelings of fear, anxiety or insecurity can evoke psychological defence mechanisms and reactance. These defence mechanisms can counteract the intended effect of the message [38] and can take various forms, such as denial (“That’s not true!”), ridicule (“That’s absurd!”), neutralization (“That won’t happen to me!”) or minimization (“That’s all terribly exaggerated!”). They reduce the perceived fear and ensure that the message is not taken seriously [41].

### Attitude, social norms and perceived behaviour control – the theory of planned behaviour

Within the P.A.R.T.Y. programme information about types of injury, their consequences or care processes are conveyed to the participants. This information may also change the participant’s behaviour. The Theory of Planned Behaviour (TBP) developed by Ajzen describes how cognitive beliefs and the change thereof can influence behaviour [43]. This theory is well established and often applied in the context of traffic behaviour (e.g. [44–46]). According to the theory of planned behaviour, rational decision-making processes explain human behaviour. Actual behaviour is determined by its intention to carry out the behaviour. Intention is in turn determined by three components: the attitude towards this behaviour, the subjective norm and perceived behavioural control towards this behaviour. Attitude results from previous positive and negative experiences with the behaviour (behavioural beliefs). Subjective norm describes the perceived pressure to comply with the expectation from relevant others (normative beliefs) concerning the certain behaviour. Norms play a special role here, since the P.A.R.T.Y. programme is a group intervention for an entire school class. Each participant learns in one way or another how his/her classmates think about risk-taking behaviour, what they think is wrong or right, or what they consider to be appropriate or desired behaviour in this context. These so-called social norms influence behaviour as well [47]. Perceived behavioural control represents the perceived scope of action, i.e. how easy or difficult a person finds it to show a certain behaviour. It is influenced by various factors and conditions (control beliefs). Perceived behavioural control is a key factor, as it not only influences behaviour indirectly via the intention but also directly [43].

## Methods

### Study area

P.A.R.T.Y. is a setting-based prevention approach to reach young people in a part of their living environment, namely the school. The program itself is implemented in clinics. Thus, the evaluation study had to be conducted in collaboration with schools and clinics. Furthermore, this is a group-based intervention, since the pupils participate in P.A.R.T.Y. together with their classmates. Therefore, they are considered as members of a cluster (the class) rather than individuals. In the school year 2016/17, a total of 19 P.A.R.T.Y. days were included in cooperation with seven trauma centres and twelve schools in five German federal states (Baden-Württemberg, Bavaria, Lower Saxony, Northrhine-Westphalia and Saxony).

### Study design and data collection

A quasi-experimental study was conducted with three measurement times. Nineteen P.A.R.T.Y. days were examined in seven different trauma centres. Twelve schools took part with 19 intervention classes. Eleven parallel classes served as a control group. A random allocation of classes to an intervention and a control group was intended, but only possible in one school. Likewise, not every school was able to provide a control class. Therefore, there was a simple one-group pre-post-test design without control group for eight of the 19 P.A.R.T.Y. days. These differences in the design were accounted for in the analysis.

The surveys were carried out consistently in the classrooms of the participating classes. The questionnaires were usually handed out personally by project staff and then collected again after completing. In this way a high response rate was ensured and the loss of completed questionnaires (e.g. by post) was prevented. In some cases, the survey was carried out by teachers if requested by the school for organizational reasons. The baseline survey (T0) took place immediately before the P.A.R.T.Y. intervention, usually the day before the intervention. The post survey (T1) was usually conducted 1 day after each P.A.R.T.Y. intervention. For the follow-up (T2), participants completed the questionnaire four to 5 months after the P.A.R.T.Y intervention. All methods were approved by an Ethics Committee and performed in accordance with relevant guidelines and regulations. An informed consent was obtained from a parent and/or legal guardian.

### Measurements

The questionnaire contained scales measuring the effects of the P.A.R.T.Y. programme, namely self-reported behaviour in traffic. Following the Generic Error Model System [48–53], items were developed that reflect the three behaviour scales “protective behaviour”, “violations” and “dangerous play”. Due to considerations of an empathic approach of action, a further behavioural scale was constructed to measure “prosocial behaviour” in road traffic. Furthermore, the questionnaire contained scales representing the assumed impact mechanisms of behaviour change described above. These included behavioural intention as a major determinant, but also items about perceived fear, severity and susceptibility towards traffic injuries (fear appeals approach), attitude, self-efficacy and social norms (Theory of Planned Behaviour). Each item was measured on a five-point-rating-scale.

The questionnaire was extensively pretested and revised. Table 1 shows the final number of items, internal consistencies, mean values and standard deviations for each scale at the three measurement scales. Most scales show acceptable (α > .70) to good (α > .80) cronbach’s alpha values. Insufficient internal consistencies (α < .60) were measured for the two scales “prosocial behaviour” and “self-efficacy” for the baseline survey (T0). They could have been increased with more items per scale. However, the overall length of the questionnaire was restricted in order not to overburden the pupils.

**Table 1:** Scales, internal consistencies, means and standard deviations at the 3 survey points

Scale	Number of items	T0		T1		T2	
		α	M (SD)	α	M (SD)	α	M (SD)
Prosocial Behaviour	3	0.51	4.01 (0.79)	0.65	4.04 (0.81)	0.61	4.00 (0.80)
Violations	3	0.65	1.49 (0.75)	0.74	1.55 (0.80)	0.72	1.51 (0.76)
Dangerous Play	4	0.75	1.81 (1.01)	0.77	1.83 (0.96)	0.74	1.81 (0.98)
Protective Behaviour	3	0.78	4.32 (1.03)	0.81	4.28 (1.00)	0.80	4.23 (1.03)
Intention	3	0.85	3.98 (0.85)	0.89	4.05 (0.89)	0.85	4.00 (0.83)
Attitude	3	0.76	4.49 (0.63)	0.80	4.52 (0.64)	0.82	4.48 (0.66)
Self-efficacy	3	0.58	3.96 (0.71)	0.65	3.92 (0.76)	0.60	3.88 (0.72)
Descriptive Norm	2	0.79	3.67 (0.86)	0.86	3.80 (0.88)	0.84	3.76 (0.86)
Injunctive Norm	2	0.72	4.26 (0.82)	0.77	4.25 (0.81)	0.77	4.23 (0.79)
Fear	2	0.72	2.60 (1.10)	0.81	2.80 (1.15)	0.75	2.67 (1.10)
Perceived Severity	2	0.66	4.00 (0.85)	0.79	4.33 (0.88)	0.74	4.30 (0.81)
Perceived Susceptibility	2	0.79	2.29 (0.94)	0.82	2.32 (0.93)	0.82	2.31 (0.91)

α= cronbach’s alpha, *M* Mean value, *SD* Standard Deviation

### Participants

In total 799 pupils took part, 574 of them at all three measurement points. The sample characteristics are shown in Table 2. Just over half of them (57.5%) belonged to the intervention group (school classes participating in the P.A.R.T.Y.) the other half to the control group. Nearly half of them were male with no differences between intervention and control group. The average age of the participants was 15.5 years (SD = 0.8). The significant difference in age is because in two schools, classes at higher grade participated in the PARTY programme, but there was no respective control group. A mean value comparison without these particular schools did not show a significant age difference. More than two-fifths of the pupils described their place of residence as a village. Slightly more than one fifth said they lived in a medium-sized or small town, the remaining 11% in a large city. The average value of the self-reported willingness to take risks was about 3.0 (SD = 1.0), with 1 corresponding to “not at all willing to take risks” and seven to “a very high willingness to take risks”. Apart from age, there were no significant differences in socio-demographic characteristics between the intervention and control groups.

**Table 2:** Sample characteristics by intervention and control group at baseline (T0)

Characteristic	Total	P.A.R.T.Y.	Control	Test value
**Sample (% [N])**	100 (574)	57.5 (330)	42.5 (244)	/
**Sex % (% [N])**				
*Male* *Female*	49.5 (284) 50.5 (290)	49.1 (162) 50.9 (168)	50.0 (122) 50.0 (122)	χ^2^ = 0.05
**Age (M [SD])**	15.5 (0.8)	15.6 (1.0)	15.4 (0.7)	t = −2.93**
**Residence (% [N])**				
*Village* *Small town* *Medium sized town* *Large town*	43.7 (249) 23.3 (133) 21.6 (123) 11.4 (65)	45.5 (150) 23.3 (77) 20.6 (68) 10.6 (35)	41.3 (99) 23.3 (56) 22.9 (55) 12.5 (30)	χ^2^ = 1.34
**Willingness to take risk (M [SD])** *(1 = not at all; 7 = very high)*	2.92 (.99)	2.93 (1.04)	2.91 (.92)	t = −.30

*M* Mean, *SD* Standard Deviation; **p < .01

### Data analyses

To determine the effectiveness of the intervention the data analysis was supposed to calculate the average effect size for the intervention groups compared to the control groups. By doing this, two characteristics of the study needed to be taken into account.

First, the data obtained from the participants are not independent because they participated as school classes (clusters). The same applies to the participants of the control group. Consequently, the statistical dependency caused by analysing clustered data has to be taken into account. Otherwise, the standard error of the calculated effect sizes is biased. Second, the P.A.R.T.Y. days took place in different clinics and were therefore not a standardized intervention. The implementation of the intervention in the daily hospital procedures, different persons involved in the programme as well as the characteristics of the participating classes are components, which produce an unavoidable heterogeneity of the P.A.R.T.Y. days between the clinics, but also partly within the same clinics. Therefore, based on the data, there is in fact a 19-fold study replication of the P.A.R.T.Y. intervention, which is always similar in structure, although locally differing. Thus, an average effect size includes different implementation contexts, which in turn generate further variance.

Therefore, a meta-analytical approach was chosen, where each P.A.R.T.Y. day was regarded as an intervention in its own right. That is, we estimated for each included P.A.R.T.Y. day its own effect size and calculated the average effect size across all 19 individual P.A.R.T.Y. days by means of a meta-analysis. This overall mean effect size represents the average of the true effects within the sample. This approach allows for handling the expected variability of effect sizes and the uncertainty of estimates (confidence intervals) appropriately. It also allows for taking the different sample sizes and study designs of each P.A.R.T.Y. day into account.

In a first step, we began by carrying out descriptive analyses: For each of the 19 P.A.R.T.Y. days, mean value differences and their standard deviations for intervention and control group were calculated between the baseline and post-intervention survey as well as between baseline and the follow-up survey. From the descriptive data, the effect size Hedges’ g was calculated for each measuring instrument and each P.A.R.T.Y. day according to Borenstein et al. [54]. Statistically, the effect strength has the same meaning regardless of the differences in study designs of each P.A.R.T.Y. day evaluation. Afterwards, a meta-analytical synthesis based on a random effects model (also according to Borenstein et al. [54]) was performed for each construct assessed in the questionnaire.

In further analyses, the Mplus computer programme was used for estimating path models. These models describe possible relations between the scales explaining the impact of P.A.R.T.Y. on pupils’ self-reported behaviour. It follows the potential impact mechanisms introduced in chapter 2. Therefore, four models were estimated and compared. To reduce the complexity of the models we reduced the four dependent behavioural measurements to two constructs: approved behaviour (prosocial behaviour and protective behaviour) and disapproved behaviour (violations and dangerous play). Independent path models for both study waves were estimated instead of conducting cross-legged panel analysis to reduce complexity. The models include dependent mean differences of many variables at once, including the influence of participation in P.A.R.T.Y. as a dummy variable.

## Results

### The effectiveness of the P.A.R.T.Y. intervention

**Fig. 1:**
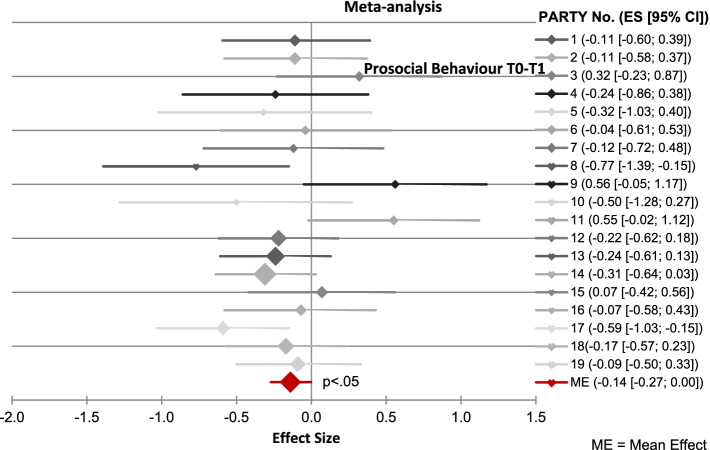
Meta-analytical result (forest plots) using the example of “prosocial behaviour” between the first (T0) and second (T1) measurement time

By transferring the effect sizes into forest plots the variation of the single effect sizes became apparent, confirming the choice of our analysis strategy. For example, in Fig. 1 the effect sizes of prosocial behaviour change from baseline (T0) to post intervention (T1) range from −.77 (P.A.R.T.Y. day No.8) to +.56 (P.A.R.T.Y. day No.9). Negative values mean a positive change in the scale, as the mean value of the post intervention (T1) was subtracted from the mean value of the baseline (T0). This applies to all scales, except those with a negative connotation (violations and dangerous play). For these measures, a negative result represents a negative change. The magnitudes of the effect sizes are interpreted, following Cohen [55]: 0.2 to 0.4 = small effect, 0.5 to 0.7 = medium effect and > 0.8 = large effect. For the change of “prosocial behaviour” between T0 baseline and T1 post intervention wave the random effects model across all 19 P.A.R.T.Y. interventions show a statistically, however small, average effect size of g = − 0.14 (95%CI = − 0.27 – 0.00). This indicates a small increase in prosocial behaviours after participating in the P.A.R.T.Y. programme day at T1.

Table 3 shows the mean effect sizes calculated for all model constructs in the same way as reported for prosocial behaviour in Fig. 1 by comparing the T0 baseline and T1 post intervention data as well as the T0 baseline and T2 follow up data. Overall, there were statistically significant but small effect sizes for the T0-T1 change in the constructs prosocial behaviour and protective behaviour. There are no statistically significant effect sizes for disapproved behaviour violations and dangerous play. Furthermore, there are small, statistically significant mean effect sizes for a number of potential determinants of impact, such as intention, descriptive norm, perceived severity etc. A medium effect size could only be identified for the perceived severity (ES_mean_ = −.61). Thus, the P.A.R.T.Y. intervention most strongly affects pupils’ perception of the severity of accident injuries. It is noticeable that P.A.R.T.Y. obviously has no effect on cognitive processes like behavioural attitude and self-efficacy respective behaviour control. Likewise, P.A.R.T.Y. seemed to affect the perception of which behaviours one’s peers are typically performing (descriptive norm), but not of which behaviours are approved or disapproved by these peers (injunctive norm).

**Table 3:** Meta-analytical results of the behaviour dimensions and their potential determinants for both survey periods

	Measurements	Mean Effect Size (ES_**mean**_)	
		T0-T1	T0-T2
Behaviour Dimensions	Prosocial Behaviour	−0.14*	n.s.
	Violations	n.s.	n.s.
	Dangerous Play	n.s.	n.s.
	Protective Behaviour	−0.20**	n.s.
Determinants	Intention	−0.23**	n.s.
	Attitude	n.s.	n.s.
	Self-efficacy	n.s.	n.s.
	Descriptive Norm	−0.24**	n.s.
	Injunctive Norm	n.s.	n.s.
	Fear/Threat	−0.20**	n.s.
	**Perceived Severity**	−0.61**	−0.37**
	Perceived Susceptibility	−0.12*	n.s.

T0-T1 = 1st survey period; T0-T2 = 2nd survey period; * = *p* < .05; ** = *p* < .01; n. s. = not significant

However, comparing the T0 baseline data with the T2 follow-up data – collected four to 5 months after the P.A.R.T.Y. days – there is only one statistical mean difference remaining. Only the perceived severity of accident injuries still shows a small positive effect in the long term (ES_mean_ = −.37).

### The evaluation of impact models to explain behaviour change

In the short term, the meta-analysis proves small, statistically significant effects of the P.A.R.T.Y. programme on self-reported approved behaviour (prosocial and protective) and behavioural intentions. In the medium term, however, only one of the effects remains significant. Hence, the aim of the following path models was to identify possible explanations for the effects that occurred in the short term but did not last.

The correlation matrices of the mean scale values at all three survey points are presented in the Supplementary Tables 1, 2 and 3. With few exceptions, almost all variables correlated significantly with each other - depending on the alignment - either positively or negatively as expected. In these cross-sectional associations, it becomes clear that cognitive variables such as attitude, norms and self-efficacy correlate more strongly with behaviour and behavioural intention than the emotional ones.

### The influence of fear appeals

**Fig. 2:**
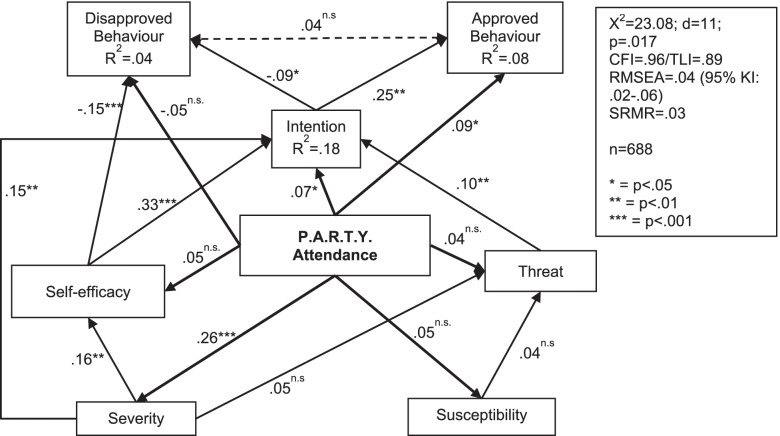
Path model of threat-related characteristics for the first survey period (T0-T1)

Based on the mean differences between the T0 and T1, respective T2 path models were estimated. Figure 2 shows the path model for the threat-related variables, its relation to whether or not pupils attend the intervention and self-reported behaviour. Following Witte’s Extended Parallel Process Model [38], the model assumes that perceived threat and self-efficacy both influence behaviour. The subjectively perceived extent of the threat, together with a person’s confidence in his or her ability to counter this threat actively, determines behaviour. Therefore, perceived severity and susceptibility of accidents were modelled as predictors of the feeling of threat in road traffic. The threat as well as the self-efficacy were modelled as determinants of behavioural intention. The bold arrows show the effect of participating in the P.A.R.T.Y. programme.

As Fig. 2 shows, the data cannot confirm that an increased perception of the severity and susceptibility also increased the feeling of threat (β = .05 and β = .04). However, the perceived severity of accidents has a direct effect on the mean change of behavioural intention and self-efficacy, i.e. an increased severity determines an increased self-efficacy and intention toward the behaviour. The results of the path analysis confirm the theoretical expectation that self-efficacy is a significant predictor of behavioural intention (ß = .33), which confirms the role of self-efficacy as a central target construct. Overall, the variables included in the model, together with the intervention effect of the P.A.R.T.Y. attendance, explain 23% of the variance of the change in intention, 4, and 8% of the change in self-reported behaviour.

It is noticeable that not in all cases the statistical intervention effects of P.A.R.T.Y. in the path models correspond to the meta-analytical results presented above. For example, the path model (Fig. 1) shows an effect of P.A.R.T.Y. attendance on perceived severity of ß = .26, which corresponds to an effect size of about g = .65. That is somewhat larger than in the meta-analytical findings (g = .61). One possible factor could be suppressor effects in multivariate analyses (e.g. [56]) which make an association between two variables stronger if the impact of third variables is controlled.

**Fig. 3:**
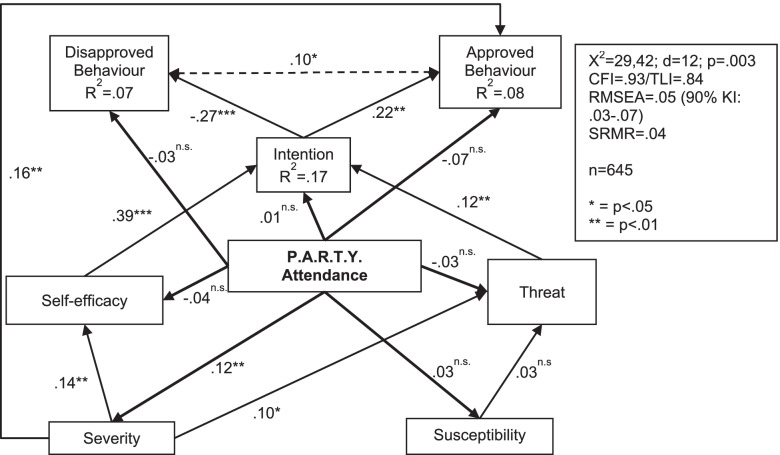
Path model of threat-related characteristics for the second survey period (T0-T2)

The model for the T0-T2 period showed a similar picture (Fig. 3), even with a direct effect of the perceived severity level on positive behaviour (β = .16). Self-efficacy is again the most important predictor for the behavioural intention (β = .39). The two model fits are acceptable, but only when taking into account the direct impact paths of the perceived severity on intention and behaviour. Here the variance explanation of the dependent variables lies between 7 and 17%.

In summary, the results show that the intervention does not directly increase feelings of threat. However, P.A.R.T.Y. obviously affected a theoretical determinant of the threat, namely the perceived severity whose change is directly related to a change in approved behaviour. Nevertheless, in turn it does not increase a person’s confidence in his or her ability to counter this threat actively (self-efficacy). This already provides one possible explanation for why the P.A.R.T.Y. does not have the intended effects on behaviour.

### The importance of the TPB constructs

**Fig. 4:**
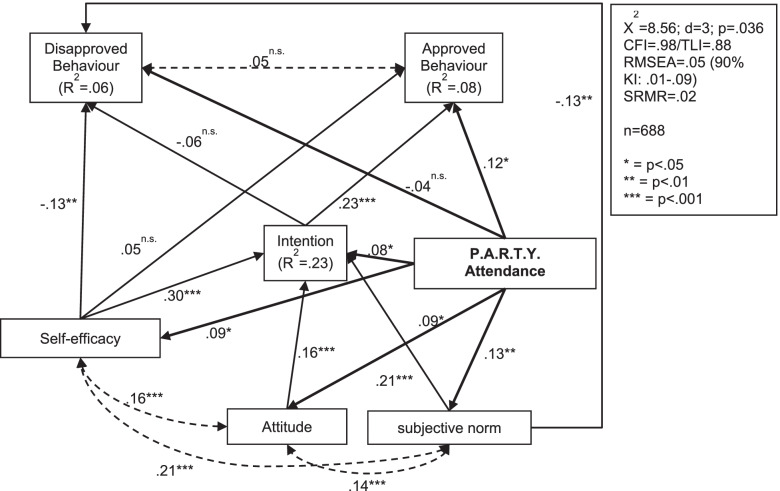
Path model of cognitive beliefs related characteristics (TPB) for the first survey period (T0-T1)

Figure 4 shows the results of a path model testing the structural relations postulated by Ajzen’s theory of planned behaviour [43]. The theory states that attitude, subjective norm and self-efficacy determine the behavioural intention, which in turn predicts behaviour. The correlation matrixes show that self-efficacy, attitude and subjective norm correlate as expected (see Supplementary Tables 1, 2 and 3). Similarly, in Fig. 3 higher mean difference in all three variables also predict a positive change in behavioural intention. This means the more self-efficacy, attitude and subjective norm of adhering to traffic rules increases from T0 to T1, the more increased the intention to adhere will be in the same period. Together with the effect of participating in P.A.R.T.Y., the three predictors explained 23% of the variance of the change in intention. The strongest influence was found for self-efficacy (β = .30; *p* < .001). The intention in turn affected approved behaviour, but not disapproved behaviour. Conversely, the direct effect of self-efficacy on behaviour assumed in theory can only be proven for disapproved behaviour. The theoretically assumed model does not fit the data well. In order to achieve a better model fit, the direct relation between subjective norm and disapproved behaviour was added to the model even though it is not included in the theory.

**Fig. 5:**
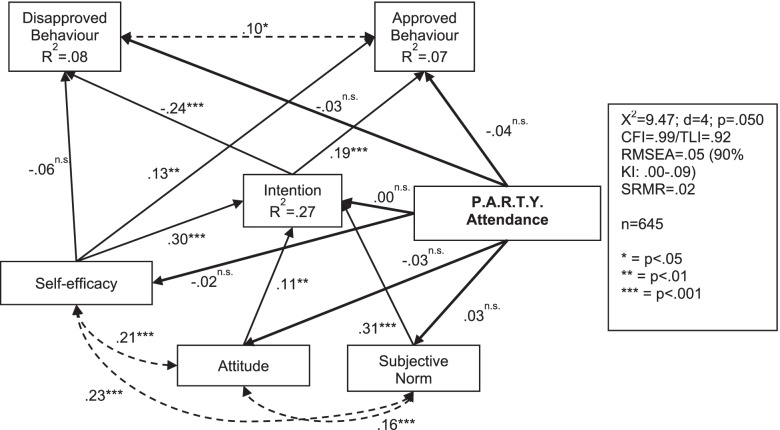
Path model of cognitive beliefs related characteristics (TPB) for the second survey period (T0-T2)

The data of the second survey period T0-T2 reflect the Theory of Planned Behaviour somewhat better (Fig. 5). This model describes the effect of the programme four to 5 months later. The rather good model fit shows that the data fit the assumed effect paths. An observed positive mean difference in the behavioural intention predicts an even stronger expression of both behavioural criteria. This means that an increased intention to follow traffic rules from T0 to T2 is accompanied by less self-reported disapproved and more self-reported approved behaviour in traffic. The importance of self-efficacy for the behavioural intention is also apparent here. The more the students are convinced that it is not a problem for them to follow traffic rules, the higher is their intention to carry out this behaviour. Whether or not pupils participated on the intervention did not influence self-reported behaviour. This confirms the results of the meta-analysis. Furthermore, in the path model there are no significant relationships to self-efficacy, attitude, subjective norm or intention. This means that the P.A.R.T.Y. programme does not have a lasting effect on the mechanisms of action according to the Theory of Planned Behaviour.

In both models, the measured changes in the predictors from the Theory of Planned Behaviour can explain more than 20% of the variance of the change in intention.

## Discussion

The aim of the present study was to evaluate the effectiveness of the P.A.R.T.Y. programme in Germany. The main results can be summarized as follows:

- Immediately after the intervention, small positive effects could be shown for most of the parameters. These effects could not be observed in the medium term and especially not for self-reported behaviour.
- Both in the short and medium term, the results showed a significant effect only on the threat-related characteristic perceived severity of accidental injuries.
- In the path analyses, the predictive influence of cognitive beliefs in particularly on a change in behavioural intention could be confirmed, such as self-efficacy or social norms. However, the P.A.R.T.Y. programme had no or only short-term effects on these factors.

Despite the worldwide dissemination of the P.A.R.T.Y. programme, there are only few methodologically sound studies about its effectiveness and no study investigating the impact mechanism by which the behaviour change is supposed to be achieved. Even though the findings of the international evaluation studies are difficult to compare due to different methodology, this P.A.R.T.Y. evaluation study showed similar results with regard to behaviour-related outcomes. We found small effects for self-reported behaviour immediate after the P.A.R.T.Y. intervention. This is in line with studies whose study period was rather short and lasted at most up to 1 month after the intervention [19, 21, 22]. We did not find long-term behavioural effects which is in line with the findings of Barnes and MacGregor [36]. There were also no changes in behaviour 12 months after the intervention. In contrast Wheeler and Mackelson (2009) found effects on self-reported behaviours such as cell phone use while driving or speeding 12 and 24 months after program implementation [22].

In contrast to the lack of efficacy with regard to attitude in the present study, some studies report at least short-term effects [17, 18, 57]. On the other hand, Barnes and MacGregor (2010) found no change in attitudes toward risk behaviours after 1 year in their study [36].

Whether international P.A.R.T.Y. program versions achieve a change in other behavioural determinants like social norms or self-efficacy remains unclear, as these factors has not been included in previous effectiveness studies.

Our results show that it seems unlikely that the P.A.R.T.Y. intervention programme in its current form will achieve its goal of reducing the risk behaviour of young people in traffic. An important reason for this seems to be that the assumptions on which the programme is based are insufficient, namely that young people can be motivated to change risky behaviour by strong emotions such as fear. Fear appeals may arouse the interest and attention of young people as a kind of “door opener” but without strengthening psychosocial beliefs namely self-efficacy, they seem to be more a “flash in the pan”. Thus, the PARTY program seems to be classified in a series of educational measures to change behaviour, which produce short-term effects, but for which no behaviour-related effects can be proven in the long term [10, 12, 13].

Following the debate on fear appeals mentioned at the beginning of this paper, the models of the threat components nevertheless showed that perceived severity is able to influence self-efficacy, behavioural intention and even behaviour. Thus small, short-term effects can be achieved by communicating fear appeals. But the conditions for this should be further researched. Furthermore, according to Witte [38], in combination with the increased perception of severity the reported low self-efficacy may even lead to undesired behavioural strategies such as defaming the threat. The results support the suggestion of Kok et al. [27] that self-efficacy must be taken into account as a central component in the successful long-term application of fear appeals for behaviour change. That indicates a more complex impact model of P.A.R.T.Y. in which cognitive beliefs such as self-efficacy and social norms seem to play an important role. For a long-term change in behaviour, the experiences in the clinic must obviously be processed cognitively. For example, they need to derive options of actions for themselves and strengthen their belief, so that they can avoid or reduce risky behaviour. To make the program more effective the previously assumed mechanisms of action should be revised and/or replaced by a theory-based program model. This program model should contain essential psychological components to achieve a change in the behaviour of young people. It should also be the basis for the systematic revision and further development of the P.A.R.T.Y. programme.

### Practical implications

The results indicate that there needs to be elements in the programme to strengthen the cognitive beliefs in young people of avoiding risk behaviour in traffic. According to Bandura [58], a lasting belief in self-efficacy results from gaining positive experiences after successfully overcoming problems and with persistent, determined effort. Experience in dealing with problems provides the necessary tools for self-efficacy: accurate cognitive assessment of the degree of difficulty, stamina and self-regulation (self-motivation, self-soothing, etc.). For example, during the P.A.R.T.Y. programme, students could be confronted with challenges in role-plays in which they have to recognize dangerous situations in traffic as well as learn and apply concrete measures to cope with these situations.

The social norms approach (e.g. 59) could be used to influence descriptive norms regarding risk behaviour in the peer group. The approach is based on scientific evidence that suggests that students often overestimate the substance consumption of their peers. This overestimation can lead to an increase in their own consumption [59, 60]. This describes the misjudgement of a situation by believing that the majority accepts certain norms or behaviours when the majority secretly rejects them. Interventions based on the social norms approach can present the actual norm and correct the misjudgement of peer consumption and in turn change one’s own behaviour. The P.A.R.T.Y. programme focuses on deviations from the social norm and their consequences, such as traffic rule violations and resulting accidents and injuries. This may lead to the false impression of young people that traffic rule violations are the norm in their peer group. According to the social norm approach, this belief can be changed by making transparent to the young people that the silent majority of their peer group actually adheres to rules.

### Limitations

We used a meta-analysis based on a random effects model to control the sources of variance within and between the classes or clinics that participated in the study. However, this method does not control bias at the beginning of data collection, namely in the selection of study units. The data are only based on a quasi-experiment, which means the school classes have been chosen by the schools for both intervention and control group. Preferably, the classes in all schools would have been assigned randomly to either the control or the intervention group (so called cluster-randomized study design). As this was only possible in one school, the equivalence of control and treatment conditions before the intervention could not be fully ensured as indicated by statistically significant age differences between school classes before the intervention.

The reliability of some of the measurement scales is rather low, especially the reliability of the self-efficacy scale. Since the reliability of measuring instruments depends not least on the number of items used to measure a construct, there is often a conflict between reliability (i.e. number of item) and feasibility (overall length) of the questionnaire in intervention studies. The questionnaire could not exceed a certain length to be acceptable for students and schools. Therefore, we had to limit the number of items used to measure each construct. Because of the lack of an explicit programme theory, we needed to include a broad set of constructs from different theoretical models. That left only few items per construct. For self-efficacy, for example, there are various dimensions discussed in the literature [58]. The low reliability of the self-efficacy scale probably reflects the fact, that the few items in the questionnaire did not tap all subdimensions of self-efficacy. Nevertheless, the results concerning the role and importance of self-efficacy are unambiguous.

## Conclusions

Summarising the results from this study the following main conclusions can be drawn. Apparently, communicating threat alone is not sufficient to achieve a lasting change in self-reported traffic-related risk behaviour. In addition, it needs elements to change cognitive beliefs as well. In the short term, such elements should be developed in a theory-based manner. Most promising for behaviour change are elements enhancing self-efficacy or eliciting descriptive social norm. Cognitive processing could be facilitated with elements carried before and after the actual P.A.R.T.Y. day at schools, e.g. preparation material, refresher courses. An explicit programme theory should be developed and tested that could guide the revision of the programme. Meta-analytical procedures have proven to be appropriate for programme evaluations where differences across implementations are typical for the programme rather than an indication of inadequate implementation. There are randomized study designs and statistical procedures available not only on an individual level but for clusters (e.g. school classes). They allow for less biased effects, which gives a better picture of the effects and underlying impact mechanism of programme interventions.

## Supplementary Information

**Additional file 1: Supplementary Table 1.** Correlation matrix of the mean scale values to the baseline survey (T0).

**Additional file 2: Supplementary Table 2.** Correlation matrix of the mean scale values to the post survey (T1).

**Additional file 3: Supplementary Table 3.** Correlation matrix of the mean scale values to the follow-up (T2).

## Data Availability

Please contact Tina Gehlert (t.gehlert@gdv.de) for data requests.

## Abbreviations

P.A.R.T.Y.: Prevent Alcohol and Risk-Related Trauma in Youth
TBP: Theory of Planned Behaviour
