# Supplementary material for: Change of risk behaviour in young people – the effectiveness of the trauma prevention programme P.A.R.T.Y. considering the effect of fear appeals and cognitive processes
Source: BMC Public Health. 2022 Mar 26;22:595. doi: 10.1186/s12889-022-12918-2 (PMC8962068; doi:10.1186/s12889-022-12918-2)
Supplement: Supplementary file 1 — Additional file 1: Supplementary Table 1. Correlation matrix of the mean scale values to the baseline survey (T0). [file 12889_2022_12918_MOESM1_ESM.docx]

**Supplementary table 1: Correlation matrix of the mean scale values to the baseline survey (T0)**

| **Scale** | **(1)** | **(2)** | **(3)** | **(4)** | **(5)** | **(6)** | **(7)** | **(8)** | **(9)** |
| --- | --- | --- | --- | --- | --- | --- | --- | --- | --- |
| (1) Approved  Behaviour | 1 |  |  |  |  |  |  |  |  |
| (2) Disapproved  Behaviour | -.17** | 1 |  |  |  |  |  |  |  |
| (3) Intention | .45** | -.45** | 1 |  |  |  |  |  |  |
| (4) Attitude | .31** | -.32** | .54** | 1 |  |  |  |  |  |
| (5) Subjective Norm | .32** | -.29** | .58** | .39** | 1 |  |  |  |  |
| (6) Self-efficacy | .38** | -.29** | .61** | .44** | .47** | 1 |  |  |  |
| (7) Fear | .14** | -.12** | .24** | .16** | .14** | .15** | 1 |  |  |
| (8) Severity | .15** | -.01* | .24** | .21** | .21** | .20** | .09* | 1 |  |
| (9) Susceptibility | -.10** | .17** | -.13** | -.08* | -.15** | -.18** | .07* | .02 | 1 |

**Correlation is significant at the 0.01 level (2-tailed).

*Correlation is significant at the 0.05 level (2-tailed).
