# Supplementary material for: Change of risk behaviour in young people – the effectiveness of the trauma prevention programme P.A.R.T.Y. considering the effect of fear appeals and cognitive processes
Source: BMC Public Health. 2022 Mar 26;22:595. doi: 10.1186/s12889-022-12918-2 (PMC8962068; doi:10.1186/s12889-022-12918-2)
Supplement: Supplementary file 2 — Additional file 2: Supplementary Table 2. Correlation matrix of the mean scale values to the post survey (T1). [file 12889_2022_12918_MOESM2_ESM.docx]

**Supplementary table 2: Correlation matrix of the mean scale values to the post survey (T1)**

| **Scale** | **(1)** | **(2)** | **(3)** | **(4)** | **(5)** | **(6)** | **(7)** | **(8)** | **(9)** |
| --- | --- | --- | --- | --- | --- | --- | --- | --- | --- |
| (1) Approved  Behaviour | 1 |  |  |  |  |  |  |  |  |
| (2) Disapproved  Behaviour | -.32** | 1 |  |  |  |  |  |  |  |
| (3) Intention | .61** | -.49** | 1 |  |  |  |  |  |  |
| (4) Attitude | .47** | -.42** | .65** | 1 |  |  |  |  |  |
| (5) Subjective Norm | .51** | -.35** | .63** | .50** | 1 |  |  |  |  |
| (6) Self-efficacy | .49** | -.33** | .63** | .53** | .54** | 1 |  |  |  |
| (7) Fear | .14** | -.12** | .23** | .19** | .12** | .17** | 1 |  |  |
| (8) Severity | .29** | -.20** | .34** | .33** | .32** | .30** | .13* | 1 |  |
| (9) Susceptibility | -.08* | .12** | -.11** | -.09* | -.13** | -.12** | .12* | .03 | 1 |

**Correlation is significant at the 0.01 level (2-tailed).

*Correlation is significant at the 0.05 level (2-tailed).
