# Supplementary material for: Change of risk behaviour in young people – the effectiveness of the trauma prevention programme P.A.R.T.Y. considering the effect of fear appeals and cognitive processes
Source: BMC Public Health. 2022 Mar 26;22:595. doi: 10.1186/s12889-022-12918-2 (PMC8962068; doi:10.1186/s12889-022-12918-2)
Supplement: Supplementary file 3 — Additional file 3: Supplementary Table 3. Correlation matrix of the mean scale values to the follow-up (T2). [file 12889_2022_12918_MOESM3_ESM.docx]

**Supplementary table 3: Correlation matrix of the mean scale values to the follow-up (T2)**

| **Scale** | **(1)** | **(2)** | **(3)** | **(4)** | **(5)** | **(6)** | **(7)** | **(8)** | **(9)** |
| --- | --- | --- | --- | --- | --- | --- | --- | --- | --- |
| (1) Approved  Behaviour | 1 |  |  |  |  |  |  |  |  |
| (2) Disapproved  Behaviour | -.23** | 1 |  |  |  |  |  |  |  |
| (3) Intention | .51** | -.46** | 1 |  |  |  |  |  |  |
| (4) Attitude | .37** | -.34** | .59** | 1 |  |  |  |  |  |
| (5) Subjective Norm | .40** | -.29** | .62** | .45** | 1 |  |  |  |  |
| (6) Self-efficacy | .41** | -.29** | .59** | .46** | .47** | 1 |  |  |  |
| (7) Fear | .17** | -.14** | .29** | .21** | .13** | .23** | 1 |  |  |
| (8) Severity | .25** | -.15** | .32** | .30** | .25** | .22** | .10* | 1 |  |
| (9) Susceptibility | -.10** | .13** | -.13** | -.11* | -.14** | -.17** | .10* | -.02 | 1 |

**Correlation is significant at the 0.01 level (2-tailed).

*Correlation is significant at the 0.05 level (2-tailed).
